# High-Quality Draft Genome Sequence of Streptomyces albidoflavus CCOS 2040, Isolated from a Swiss Soil Sample

**DOI:** 10.1128/mra.01225-22

**Published:** 2023-02-01

**Authors:** Joël F. Pothier, Valentin Bolt, Fabienne Arn, David Frasson, Nicola Rhyner, Martin Sievers

**Affiliations:** a Environmental Genomics and Systems Biology Research Group, Institute of Natural Resource Sciences, Zurich University of Applied Sciences (ZHAW), Wädenswil, Switzerland; b Microbiology and Molecular Biology, Institute of Chemistry and Biotechnology, ZHAW, Wädenswil, Switzerland; University of Southern California

## Abstract

Here, we report the high-quality draft genome sequence of the actinomycete Streptomyces albidoflavus CCOS 2040, isolated from a Swiss soil sample. The genome contains 7,136,301 bp with 73.35% GC content. In total, 22 biosynthetic gene clusters, including polyketides and terpenes, were predicted within the sequenced genome.

## ANNOUNCEMENT

In continuing bioprospecting efforts to discover new bioactive natural products, Streptomyces albidoflavus CCOS 2040 was isolated from a soil sample (ca. 10 g) collected on 12 August 2019 using a spoon from the O horizon (0 to 5 cm) of a grassland in the canton Zurich, Switzerland. This strain was isolated as described previously (1), maintained at −80°C as glycerol stocks, and deposited at the Culture Collection of Switzerland as CCOS 2040. Partial 16S rRNA and BLASTN analysis (1) revealed an identity belonging to the *Streptomyces* genus (100% identity, GenBank accession number CP047147 as closest match). An ethyl acetate extract of a CCOS 2040 growth supernatant inhibited the control strain Staphylococcus aureus subsp. *aureus* ATCC 25923 but also the methicillin-resistant S. aureus (MRSA) strain ATCC BAA-44 and the vancomycin (VanA-type) resistant Enterococcus faecium CCOS 686 strain. Cultivation of the CCOS 2040 strain in NL148sb medium, preparation of the extract, and antimicrobial testing were performed as described previously (1, 2). To further understand the biosynthetic potential of CCOS 2040, we obtained its genome sequence using a hybrid approach.

After revival on R2A agar for 7 days at 28°C, a single colony was transferred to 5 mL R2A medium and grown at 28°C with shaking at 100 rpm. The 5 mL culture was used to inoculate 100 mL of R2A medium and grown for 7 days. Genomic DNA (gDNA) was isolated using the Genomic-tip 100/G column (Qiagen) following instructions except for a final elution with water. The gDNA was checked by gel electrophoresis and quantified at 105 ng · μL^−1^ using a high-sensitivity (HS) double-stranded DNA (dsDNA) kit with a Fluo-100B fluorometer (Allsheng).

For short-read sequencing, the library preparation was done using the Nextera XT DNA library prep kit (Illumina). Sequencing was performed on an iSeq 100 instrument (paired-end; 150 bp) using iSeq 100 i1 reagent v2 (Illumina).

For long-read sequencing, 200 μL of the same gDNA extract was size selected and purified using 1× CleanNGS paramagnetic beads (CleanNA) with final resuspension in 100 μL of Tris-HCl (10 mM; pH 8.5). After quantification as above and without gDNA shearing, library preparation and sequencing were performed with a ligation sequencing kit (Oxford Nanopore Technologies [ONT]) and run on a R9.4.1 Flongle flow cell (ONT) with a MinION Mk1B sequencing device (ONT). Reads were base called using Guppy version 5.0.11.

A *de novo* hybrid assembly conducted with MaSuRCA version 4.0.6 (3) resulted in three contigs. Two of them showing higher coverage were identified to be plasmids that were found to be circular due to direct repeat sequences (overlapping ends), as also confirmed by a *de novo* hybrid assembly using Unicycler version 0.4.9 (4). The third contig was identified as the chromosome and was expected to be linear, as reported for other streptomycetes (5–8), although the typical terminal inverted repeats encompassing palindromes that form telomeric secondary structures (6, 9) could not be detected, and only hits with low homology were observed with known archetypal and nonarchetypal telomeric proteins (Tap/Tpg and Tac/Tpc, respectively). The Type (Strain) Genome Server (10) assigned the strain to the *S*. *albidoflavus* species. The genome was annotated using Bakta version 1.5.1 (11) and the database version 4.0. All tools were run with default parameters.

Different classes of biosynthetic gene clusters (BGCs) were identified using antiSMASH version 6.0.1 (12). Within 22 regions, 19 known BGCs were identified, which included terpenes, nonribosomal peptide synthetase (NRPS), and polyketides. Clusters with 100% similarity, including antimycin, ectoine, desferrioxamine B, surugamide A/D, SAL-2242, and geosmin, were detected within the sequenced genome. Deeper analysis and mining of the gene clusters are required to find novel pharmaceutically important secondary metabolites.

### Data availability.

This high-quality draft genome sequence has been deposited in ENA under BioProject number PRJEB49330. The genome and raw read accession numbers are shown in Table 1.

**TABLE 1:** Metadata, genome metrics, and accession number of the newly sequenced Streptomyces albidoflavus CCOS 2040 high-quality draft genome^*a*^

Variable	Data
Strain	CCOS 2040
Isolate	STUP19_F108
Country, yr	Switzerland, 2019
Latitude, longitude	47.392111°N, 8.656389°E
Origin	Grassland soil
Genome size (bp)	7,136,301 bp
GC content (%)	73.35
No. of CDS	6,147
No. of rRNA (5S, 16S, 23S)	21 (7, 7, 7)
No. of tRNAs	68
No. of closed plasmids	2
	
Illumina data	
Total no. of reads	10,468,188
Avg. read length (bp)	142
Avg. coverage (×)	199
	
Oxford Nanopore data	
Total no. of reads	122,306
Read length *N*_50_ (bp)	3,246
Avg. coverage (×)	26
	
SRA accession no. (iSeq/MinION)	ERR7671851, ERR7671854
ENA accession no.	OX371412 (chr.), OX371413 (p81), OX371414 (p7)
BUSCO scores (%)^*b*^	*C*: 98.4% [*S*: 98.1%, *D*: 0.3%], *F*: 0.6%, *M*: 1.0%, *n*: 1,579

a CDS, coding DNA sequences; SRA, Sequence Read Archive; ENA, European Nucleotide Archive; chr., chromosome; BUSCO, Benchmarking Universal Single-Copy Orthologs.

b Assessed with BUSCO version 5.2.2 and the streptomycetales_odb10 (2020-03-06) lineage. *C*, complete BUSCOs; *S*, complete and single-copy BUSCOs; *D*, complete and duplicated BUSCOs; *F*, fragmented BUSCOs; *M*, missing BUSCOs; *n*, total BUSCO groups searched.
